# Validation of Tracheal Sound-Based Respiratory Effort Monitoring for Obstructive Sleep Apnoea Diagnosis

**DOI:** 10.3390/jcm13123628

**Published:** 2024-06-20

**Authors:** Mireia Muñoz Rojo, Renard Xaviero Adhi Pramono, Nikesh Devani, Matthew Thomas, Swapna Mandal, Esther Rodriguez-Villegas

**Affiliations:** 1 Acurable, London SW1H 0NB, UK; matthew.thomas@acurable.com; 2 Wearable Technologies Lab, Department of Electrical and Electronic Engineering, Imperial College of Science Technology and Medicine, London SW7 2BX, UK; renard.pramono14@imperial.ac.uk (R.X.A.P.); e.rodriguez@imperial.ac.uk (E.R.-V.); 3 Thoracic Medicine, Royal Free London NHS Foundation Trust, London NW3 2QG, UK; nikesh.devani@nhs.net (N.D.); swapnamandal@nhs.net (S.M.)

**Keywords:** OSA, respiratory effort, RIP, central apnoea, obstructive apnoea, sleep apnoea

## Abstract

**Background:** Respiratory effort is considered important in the context of the diagnosis of obstructive sleep apnoea (OSA), as well as other sleep disorders. However, current monitoring techniques can be obtrusive and interfere with a patient’s natural sleep. This study examines the reliability of an unobtrusive tracheal sound-based approach to monitor respiratory effort in the context of OSA, using manually marked respiratory inductance plethysmography (RIP) signals as a gold standard for validation. **Methods**: In total, 150 patients were trained on the use of type III cardiorespiratory polygraphy, which they took to use at home, alongside a neck-worn AcuPebble system. The respiratory effort channels obtained from the tracheal sound recordings were compared to the effort measured by the RIP bands during automatic and manual marking experiments. A total of 133 central apnoeas, 218 obstructive apnoeas, 263 obstructive hypopneas, and 270 normal breathing randomly selected segments were shuffled and blindly marked by a Registered Polysomnographic Technologist (RPSGT) in both types of channels. The RIP signals had previously also been independently marked by another expert clinician in the context of diagnosing those patients, and without access to the effort channel of AcuPebble. The classification achieved with the acoustically obtained effort was assessed with statistical metrics and the average amplitude distributions per respiratory event type for each of the different channels were also studied to assess the overlap between event types. **Results:** The performance of the acoustic effort channel was evaluated for the events where both scorers were in agreement in the marking of the gold standard reference channel, showing an average sensitivity of 90.5%, a specificity of 98.6%, and an accuracy of 96.8% against the reference standard with blind expert marking. In addition, a comparison using the Embla Remlogic 4.0 automatic software of the reference standard for classification, as opposed to the expert marking, showed that the acoustic channels outperformed the RIP channels (acoustic sensitivity: 71.9%; acoustic specificity: 97.2%; RIP sensitivity: 70.1%; RIP specificity: 76.1%). The amplitude trends across different event types also showed that the acoustic channels exhibited a better differentiation between the amplitude distributions of different event types, which can help when doing manual interpretation. **Conclusions**: The results prove that the acoustically obtained effort channel extracted using AcuPebble is an accurate, reliable, and more patient-friendly alternative to RIP in the context of OSA.

## 1. Introduction

Respiratory effort monitoring plays a crucial role in assessing sleep disorders, particularly in the diagnosis and management of conditions like obstructive sleep apnoea (OSA) [1], which can have serious health consequences such as a higher risk of developing cardiovascular conditions, daytime fatigue, and impaired cognitive function [2,3,4,5]. More specifically, respiratory effort is conventionally used to visually differentiate between central and obstructive events, as part of the home and in-clinic gold standard multichannel-based diagnostic process for OSA, namely cardio-respiratory polygraphy and polysomnography, respectively. This is because OSA is characterised by the presence of respiratory effort caused by recurrent upper airway obstruction events, whereas central sleep apnoea involves a lack of effort caused by the failure of the nervous system to initiate breathing [6]. Hence, the presence or lack of respiratory effort can guide the treatment and disease monitoring decisions [7,8].

The measurement of oesophageal pressure (Pes) with a nasal cannula is the current gold standard technique for a precise respiratory effort measurement. However, in the context of OSA diagnosis, since the actual quantification of the effort is not critical, but rather the “pattern” shown by the signals is (and/or the presence or absence of it (which aids the differentiation of events)), respiratory inductance plethysmography (RIP) is what is recommended by the American Academy of Sleep Medicine (AASM) guidelines for a non-invasive semi-quantitative assessment of tidal volume as respiratory effort [9] as the gold standard. The rationale for this is that the invasiveness and discomfort associated with Pes are poorly tolerated, can affect sleep quality, and pose a significant challenge, limiting its use in non-laboratory settings [10].

RIP, however, is not without faults, since it can produce misleading results due to factors such as obesity, band location, and band displacement during sleep [11]. Furthermore, although to the best knowledge of the authors, data have not been published characterising the failure rate of RIP bands at home, several studies have calculated a failure rate of 7% and 12% when the effort bands were applied by a clinician [12] and when they were pre-fitted by a clinician in the lab [13], which can be used as a reference to conclude that this rate is probably significantly higher at home. In light of these limitations, alternative noninvasive respiratory effort monitoring methods are currently being explored [14,15,16,17,18,19,20]. 

Several studies have proposed the recording of mandibular movement signals to measure respiratory effort and have shown a significant agreement between the mandibular signals and Pes [11,15]. However the studies were conducted on small sample sizes and encountered challenges when differentiating between certain respiratory events. 

The feasibility of use of tracheal sounds to assess respiratory effort has also been previously reported, being demonstrated against Pes and RIP both in adults [18,19] and children [20]. These studies have reported high correlation values between tracheal sounds and Pes, as well as high sensitivity and specificity values when using tracheal sounds to classify abnormal respiratory events.

This paper aims to:

- Prove the reliability and utility of the respiratory effort channels extracted from tracheal sounds using AcuPebble, when compared to currently accepted methods.
- Demonstrate the agreement between acoustically obtained respiratory effort and the current gold standard effort measurement.
- Further establish AcuPebble as an accurate and reliable alternative to current respiratory effort monitoring techniques in the context of OSA.

## 2. Materials and Methods

### 2.1. Study Design

This study was carried out using previously collected data [21] from 150 patients aged between 18 and 70 who were referred for evaluation of possible OSA to the Sleep and Ventilation clinic at the Royal Free London Hospital NHS Foundation Trust (Trial registration number: NCT03544086). These data were acquired over an 8-month period spanning from November 2018 to July 2019. Demographic details and comorbidities of the participants are outlined in a previous study [21].

### 2.2. Eligibility Criteria

All adult patients were eligible to participate in the original study, except those aged 70 and above and those who were not proficient in English or had specific communication requirements. Furthermore, participants with known allergies to adhesive dressings, as well as those with physical or mental impairments that would hinder independent use of the new technology, were excluded from the study. Subjects with electronic body implants or extremely loose skin in the neck area, which could cause the device to swing with neck movement, were also not considered for participation. For further information, including power calculations relevant to the primary endpoints, please refer to the original study [21].

### 2.3. Reference Standard

A cardiorespiratory polygraphy (CR-PG) at-home system was used in this study to obtain the reference signals. The type III system used was the Embletta MPR Sleep System (Natus Medical, Middleton WI, USA) alongside the Embla Remlogic 4.0 software (Natus Medical, Middleton WI, USA). The channels included for analysis were abdominal and thoracic piezoelectric respiratory movement sensors, peripheral pulse oximetry, a nasal thermistor air flow sensor, snore detection, and body position tracking. 

This system was utilised due to its routine use in the Sleep and Ventilation clinic at the Royal Free London NHS Foundation Trust for diagnosing sleep-disordered breathing. Moreover, it is compliant with the technical adequacy requirements outlined by the AASM, and is thus considered a gold standard for ambulatory diagnosis of the disease.

The decision to use the type III domiciliary CR-PG monitor as reference for this study was driven by the intended ambulatory home testing nature of the AcuPebble SA100 device. The use of PSG was deemed non-representative of the real-world use case scenarios and is not common clinical practice in a domiciliary setting. Moreover, a later study has demonstrated a sensitivity of 92.82% and specificity of 97.14% when comparing the AcuPebble SA100 automated diagnosis against standard PSG [22].

**Figure 1:**
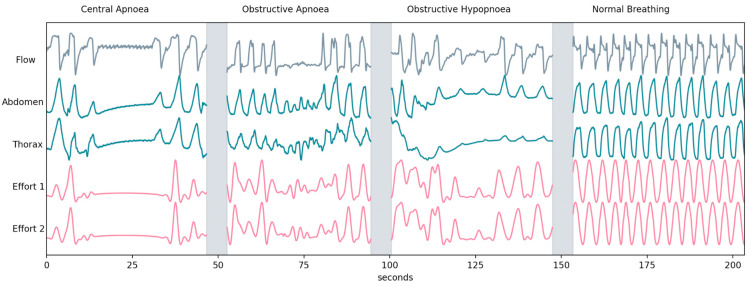
Respiratory flow, abdomen, thorax, and effort channels obtained from the acoustic signal during a central apnoea, an obstructive apnoea, an obstructive hypopnoea, and a period of normal breathing. A slight shift in the oscillations of the second effort signal can be observed with respect to the first effort signal during the obstructive apnoea event. This mirrors the paradoxical effort oscillation observed during the same event between the signals recorded with the abdominal and thoracic bands.

During the overnight studies, RIP bands were used to derive a gold standard measure of respiratory effort in the context of OSA. The tests were scored according to the AASM criteria by a team of clinicians shown in Table 1 [23]. Figure 1 shows the acoustic effort channels alongside the RIP-extracted effort during different abnormal respiratory events.

### 2.4. AcuPebble

The device used to record the tracheal sounds that were compared against the reference RIP signals used in this study was the European variant of AcuPebble SA100. This device, as previously described by Devani et al. [21], consists of a compact wearable sensor, proprietary algorithms capable of separating physiological channels and extracting relevant clinical parameters, and a fully automated diagnostic feature. Additionally, it includes a user-friendly mobile application that guides the patients through the testing process. The sensor is placed above the sternal notch on the front of the neck and secured in place by an adhesive. The test can be initiated before sleep and can be terminated upon waking by simply tapping a button on the mobile phone connected to the device. Subsequently, the collected data are uploaded to the AcuPebble SA100’s cloud platform, where it undergoes analysis using proprietary software algorithms. The resulting diagnostic output aligns with recommendations provided by the AASM.

Two channels were extracted from the AcuPebble SA100 sound recordings, representing the respiratory effort RIP signals, following equivalent physiological modelling principles as previously reported in the literature [18,24]. The performance of these channels when classifying sleep apnoea events was assessed by comparing the classification results against those achieved by the RIP thoracic and abdominal bands. In order to make this comparison, two analyses were undertaken: one based on automatic marking and one on manual marking validation.

### 2.5. Automatic Marking Validation

The agreement between the acoustic channels extracted using AcuPebble and the RIP effort channels was evaluated, to start with, by utilising an automated respiratory event marking system. Embla Remlogic 4.0 software was the tool used to identify no-effort events in the signals that would, due to the absence of respiratory exertion, correspond to central apnoeas. This software was chosen due to its compatibility with the recorded signals as the recording system used was also part of the Embletta MPR Sleep System.

Out of the 150 sleep studies, only 44 contained central apnoea events, but 4 of those 44 studies had invalid abdominal and thoracic signals (i.e., not signal), which made them unsuitable for comparison with the acoustic channel. All of the remaining 40 studies were used for the automatic validation. Limiting the comparison to just the 40 studies with valid thoracic and abdominal signals which also contained central apnoeas, as opposed to the 150, was performed for three main reasons:

1. To tackle class imbalance by trying to maximise the number of central apnoeas present in the comparison, since due to the characteristics of the population these were significantly fewer in number and appeared also in less subjects.
2. The automatic validation included a laborious manual task that entailed loading all the signals that were being compared and exporting the automatic labels achieved for both channels.
3. Additional manual validation (as described below) also took place, which increased the confidence in the results.

**Figure 2:**
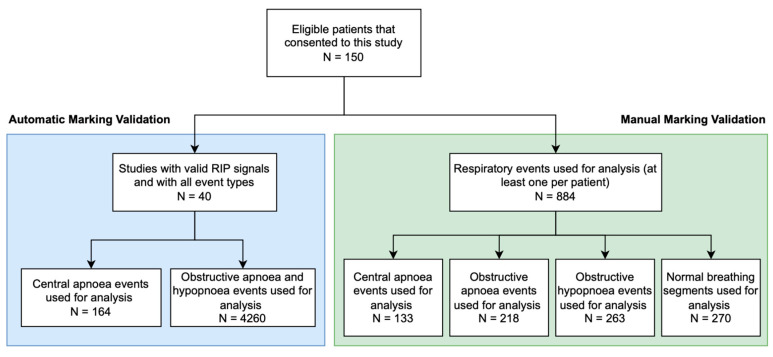
Block diagram detailing the flow of data used for analysis in both validation studies. The number of events and the type of events included in each validation analysis are shown.

The data selection process hence ensured that the 40 studies chosen were representative of the AcuPebble channel across different respiratory event types and were not influenced by selection bias. As a result of this, the data considered include a total of 164 different central events and a total of 4260 obstructive events. The two acoustic effort channels of these studies, along with their corresponding RIP effort channels, were utilised for this validation experiment. The respiratory events were located in both the acoustic channels and the RIP-extracted channel, and their timestamps were compared to the reference labels. The performance of both methods was then evaluated by statistical metrics. Figure 2 shows a summary of the data flow used for this validation analysis.

### 2.6. Manual Marking Validation

The acoustic effort channels were also blindly marked by an RPSGT certified physiologist during a manual marking trial. The scorer assessed 884 randomly presented individual events. Those events had previously been labelled by an expert clinician scorer who had marked them in the context of the study reported in [21] as central apnoeas (133), obstructive apnoeas (218), obstructive hypopnoeas (263), and normal breathing segments (270). In order to avoid bias in the results, the scorer for this study was also blinded to these values. The scorer was provided with two effort channels, a nasal flow channel, and an SpO2 channel in order to correctly be able to identify the different event types. Figure 2 shows a summary of the data flow used for this validation analysis.

These events were extracted from all 150 studies by randomly selecting at least one event of each type present in a particular study. In studies where central apnoeas were present, all central events were considered. Moreover, between one and three normal segments, obstructive apnoeas, and hypopnoea events were randomly considered per subject. The random selection of a few events per subject was carried out in order to tackle the class imbalance present in all studies and to reduce the time required to manually mark the events, while still considering all 150 subjects and event types for validation. A Python function was created to automatically randomise the event selection process described, as well as the order in which the events were assessed by the scorer for each channel in order to not introduce any bias.

The blind scorer followed the AASM criteria and marked events as central apnoea, obstructive apnoea, mixed apnoea, or obstructive hypopnoea for both the effort obtained from the acoustic signal and the RIP-extracted effort. Only the events where both scorers were in agreement were considered for validation. Statistical metrics were then applied to evaluate the classification performance of the acoustic effort channels during these events.

### 2.7. Statistical Analyses

In the first instance, a peak-to-peak signal was derived by computing the difference between the upper and lower envelopes of each respiratory effort channel. The average amplitude values present during each event were recorded and the distribution of amplitude values per event type was compared between the different effort channels. This was carried out on both to give an indication of the agreement between the channels, as well as to study the overlap between the amplitude distributions of different event types for each channel, since this would be more conducive to a visual marker confusing them.

Sensitivity, specificity, and positive and negative likelihood ratios (LR+ and LR−) alongside the 95% confidence intervals (CI) for each were the metrics used for comparison during the automatic and manual marking validation. The statistical metrics used are in accordance with those previously published and were calculated following Equations (1)–(5) [25,26]. Moreover, the results achieved can be reproduced using the University of Illinois Chicago online calculator (Diagnostic Test Calculator), where

(1) $$sensitivity=\frac{TP}{TP+FN}\times 100$$

(2) $$specificity=\frac{TN}{TN+FP}\times 100$$

(3) $$LR+=\frac{sensitivity}{1-specificity}$$

(4) $$LR-=\frac{1-sensitivity}{specificity}$$

(5) $$accuracy=\frac{TN+TP}{TN+TP+FP+FN}\times 100$$

During the automatic marking validation process, the target was the identification of apnoeas with no effort (i.e., central) in the signal. Hence, sensitivity measures the proportion of correctly identified apnoeas with no effort in the classification task, while specificity represents how well the automatic scoring system was able to avoid labelling normal breathing or apnoeas with effort (i.e., obstructive) as central apnoeas. Moreover, the LR+ represents the probability ratio between the likelihood of a true apnoea with no effort being identified and the probability of a non-central apnoea event being labelled as a central apnoea. Similarly, the LR− is the probability ratio between the likelihood of a central apnoea event not being identified and the probability of not identifying a central event when effort is present. These metrics were derived following the same criteria:

- A diagnostic output was considered a true positive (TP) when a central apnoea was identified as a no-effort event by the software;
- A false positive (FP) was identified when a central apnoea was detected during an obstructive event or during a period of normal breathing;
- A true negative (TN) output occurred when no central events were detected by the software during obstructive events or periods of normal breathing;
- Finally, a false negative (FN) occurred when no events were detected by the software during a central apnoea.

During the manual marking validation experiments, sensitivity measures the proportion of correctly classified respiratory events, while specificity indicates the proportion of events correctly classified as a different label for a specific type of respiratory event. Moreover, the LR+ represents the probability ratio between the likelihood of an event getting classified correctly and the probability of a different event type getting classified as a specific respiratory event. Similarly, the LR− represents the probability ratio of a specific event type being misclassified as another, compared to the probability of an event being classified as a different label from the specific one under consideration. These metrics were derived when considering two different classes: central and obstructive events. The criteria followed for both calculations were the same:

- A TP occurred when both the marked label (by the blind expert marker) and the reference label (as per the original expert clinicians who marked the signal in the original study, where the database originated) were in agreement. If an obstructive hypopnoea event was considered to be an obstructive apnoea event, or vice versa, the labels were considered to be in agreement;
- A diagnostic output is considered a FP when an event is marked as belonging to a specific class, but the reference label suggests it belongs to the other class;
- A TN occurs when an event that does not belong to the specified class is labelled accordingly;
- Finally, a FN output occurs when an event that belongs to the specified class is mislabelled as an event that belongs to the opposite class.

## 3. Results

### 3.1. Event Amplitude Evaluation

**Figure 3:**
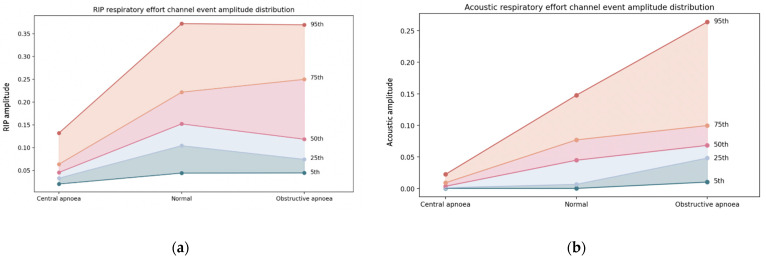
Distribution of average amplitude values of RIP effort (**a**) and acoustic effort (**b**) during normal breathing and different respiratory events. The distribution has been summarised in five centiles (5th, 25th, 50th, 75th, and 95th) for each event type.

The average amplitudes for each event were calculated and separated per event type. This was carried out to compare the differences between the amplitude distributions within channels by studying the amplitude overlap that exists between different event types for both effort channels. Although absolute values of amplitude are not important, relative differences and non-overlap help to differentiate events visually. Figure 3 displays the distribution of these amplitudes in five different centiles for the acoustics effort channel and the RIP effort channel. It can be seen how in the case of RIP there is a less clear separation in amplitude between different types of events.

### 3.2. Classification Accuracy Evaluation

#### 3.2.1. Automatic Scoring

**Figure 4:**
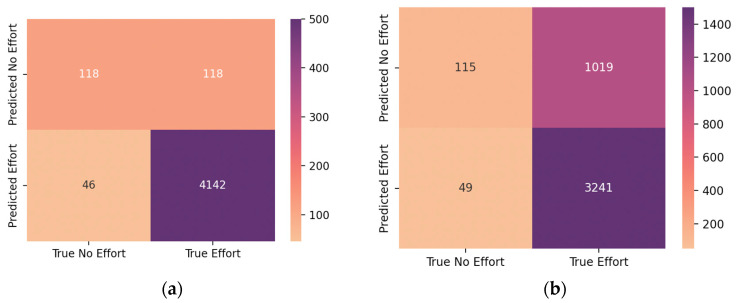
Confusion matrices for the effort channel from acoustics (**a**) and the RIP effort channel (**b**).

The statistical metrics were calculated for the classification results obtained by the automatic scoring system and are stated in Table 2. The metrics were calculated for both the no-effort classification and the correct classification of effort segments. The overall confusion matrices obtained can also be seen in Figure 4. Out of the true effort events misclassified by the Embla Remlogic 4.0 software in the effort from AcuPebble’s channel, 33.9% of them were also misclassified in the RIP-extracted effort channel. Furthermore, 31.25% of the true no-effort events that were misclassified in the acoustic channel were also misclassified in the RIP-extracted channel.

#### 3.2.2. Manual Scoring

**Figure 5:**
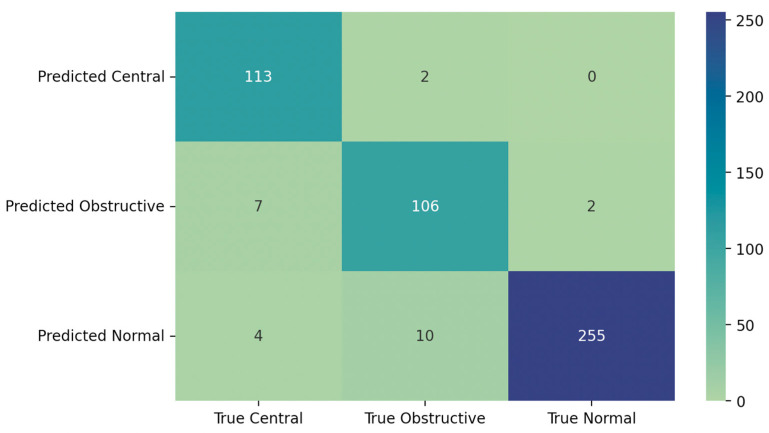
Confusion matrix showing the classification achieved by the manual marker when using the acoustic channels as a measure of respiratory effort, when considering only the events that were classified in the same way by both scorers.

The statistical metrics were calculated for two classes: a no-effort class corresponding to central apnoea events, and an effort class that corresponds to obstructive events. Table 3 displays the values derived from the classification results obtained using the effort channel from AcuPebble, only taking into consideration the events where both scorers were in agreement. The corresponding confusion matrix is shown in Figure 5.

## 4. Discussion

This study was conducted to explore the utilisation of the effort channel acoustically extracted from AcuPebble for the non-invasive assessment of respiratory effort in the context of Obstructive Sleep Apnea (OSA). The findings confirm the reliability and practicality of this method, in line with previous research that established acoustic sensing of respiratory effort as a viable alternative to thermal or pressure sensing methods [18,19,20].

Traditional techniques like Respiratory Inductance Plethysmography (RIP) often face challenges such as band displacement during sleep. The results show that AcuPebble’s effort channel achieved high accuracy, sensitivity, and specificity values when compared to the RIP channel for both the manual and automatic validations. This suggests that AcuPebble offers a promising solution that addresses these limitations by providing a less cumbersome, more user-friendly option. Such advancements could significantly lower the failure rates often seen in home-based settings and overall provide a better patient experience [12,13].

The assessment of average amplitudes for both acoustic and RIP effort channels during abnormal respiratory events and normal breathing segments showed a greater separation of values per event type in the acoustic channel. Conversely, the RIP amplitude distributions displayed a large overlap between event types, making the acoustic effort amplitude ranking more closely resemble the Pes amplitude ranking calculated in previous studies [11]. This suggests that the use of the acoustic channel to monitor respiratory effort may lead to fewer misclassifications as the amplitude differences between events are more pronounced. Moreover, the statistical metrics calculated when comparing the classification achieved between channels displayed a comparable classification performance between channels.

Despite being common practice, RIP has a tendency of underestimating respiratory effort due to factors such as lung volume and posture, causing an overestimation of central events [18,27]. This can be observed by looking at the confusion matrix resulting from the automatic marking experiments, which reveals a strong trend of RIP-extracted effort misclassifying obstructive events as central apnoeas. Nonetheless, this pattern is not as pronounced in the classification results from the acoustic channels obtained from AcuPebble, reinforcing the fact that the acoustic channel provides a better event type distinction, yielding fewer misclassifications.

During the automatic marking validation experiment, the effort channels obtained from AcuPebble showed a better performance compared to the RIP-extracted channels, achieving a higher sensitivity, specificity, and accuracy. The misinterpretation of the RIP-extracted channel by the automatic scoring system could be due to the fact that the nasal flow channel was not considered by the system, meaning that in the absence of the nasal flow, using the effort channel extracted from AcuPebble’s signals would lead to closer results to the truth when manually marked.

Although the selection process for the studies used for the automatic evaluation minimised selection bias to ensure the results would be generalizable, there is a chance the selective sampling does not fully represent specific populations. Hence, prospective validation studies in different patient populations could also be of interest to try to investigate potential differences that might not be noticeable with the used methodology.

Manual marking results showed that the acoustic effort channel presents a high accuracy when differentiating between central and obstructive events. The likelihood ratios calculated show that AcuPebble’s effort channel is able to accurately identify positive events, thereby minimising false positive misclassifications. When classifying obstructive events, the lowest sensitivity value is observed (89.8%), suggesting that there may be an overestimation of normal breathing segments that causes an increase in the number of false negative cases. However, the identification of a respiratory event relies on the correct interpretation of the nasal flow signal; the effort channel only detects the presence or lack of a respiratory effort. This, therefore, suggests that the misclassifications could be caused by the marker’s misinterpretation of the nasal flow channel.

## 5. Conclusions

In conclusion, tracheal sounds recorded non-invasively using AcuPebble can provide a highly reliable respiratory effort assessment during different abnormal respiratory effort events and normal breathing periods. The user-friendly design and affordability of this device can streamline the diagnostic process and improve accessibility for more vulnerable patients. Ultimately, AcuPebble has been previously validated against CR-PG in home environments and PSG, demonstrating that it can provide an accurate automatic diagnosis against these techniques without the need for the manual expert marking of individual channels. However, this work shows that when clinically needed, it can also allow for a non-invasive accurate monitoring of respiratory effort during sleep.

## Figures and Tables

**Table 1:** AASM guidelines for the scoring of different respiratory events. These were the criteria used to score the events during all the experiments mentioned in this paper.

Respiratory Event Type	Airflow Pattern	Respiratory Effort Pattern
Obstructive Hypopnoea	Reduction in nasal pressure of more than 30% for more than 10 s with a clear termination (strong breath and/or movement)	Increase in effort expected to start 2 or more breaths prior to event termination
Obstructive Apnoea	Decrease of at least 90% or more in the respiratory flow signal	Increase in effort begins 2 (or more) breaths prior to resumption of flow, and peaks before the peak in airflow
Central Apnoea	Same as for obstructive apnoea but no flow limitation	Absence of effort. Changes in effort synchronous with changes in flow, or the increase in effort starts 1 breath prior to resumption of flow
Mixed Apnoea	Same as for obstructive apnoea	Effort signal decreases like a central apnoea and then increases like an obstructive apnoea

**Table 2:** Performance metrics achieved during automatic marking when compared against the reference labels and considering both the RIP-extracted effort channels (left) and the acoustic effort channels (right).

Statistical Metrics	RIP Effort Channel	95% CI	Acoustic Effort Channel	95% CI
Sensitivity	70.1%	62.7% to 76.6%	71.9%	64.6% to 78.3%
Specificity	76.1%	74.8% to 77.3%	97.2%	96.7% to 97.7%
LR+	2.93	2.62 to 3.28	25.98	21 to 32
LR−	0.39	0.31 to 0.50	0.29	0.23 to 0.37
Accuracy	75.9%	74.6% to 77.1%	96.3%	95.7% to 96.8%

**Table 3:** Performance metrics achieved when using the acoustics effort channel during manual marking when compared against the reference labels for the events where both scorers were in agreement. The performance when identifying central apnoeas, as well as the performance when identifying obstructive events, was analysed.

Statistical Metrics	Central Apnoeas	95% CI	Obstructive Apnoeas	95% CI	Average
Sensitivity	91.1%	84.8% to 95%	89.8%	83.1% to 94.1%	90.5%
Specificity	99.5%	98.1% to 99.8%	97.6%	95.6% to 98.7%	98.6%
LR+	170.87	43 to 681	38.03	20 to 73	104.45
LR−	0.089	0.05 to 0.16	0.104	0.06 to 0.18	0.097
Accuracy	97.4%	95.6% to 98.5%	96.2%	93.7% to 97.2%	96.8%

## Data Availability

Data are available upon reasonable request. Data of individual participants will not be shared since consent for this has only been granted for regulatory authorities. All other information will be shared on request, provided it is not confidential for IP protection reasons. Requests should be directed to e.rodriguez@imperial.ac.uk.
